# DNA sensor cGAS-mediated immune recognition

**DOI:** 10.1007/s13238-016-0320-3

**Published:** 2016-09-30

**Authors:** Pengyan Xia, Shuo Wang, Pu Gao, Guangxia Gao, Zusen Fan

**Affiliations:** 1 Key Laboratory of Infection and Immunity of CAS, CAS Center for Excellence in Biomacromolecules, Institute of Biophysics, Chinese Academy of Sciences, Beijing, 100101 China; 2 University of Chinese Academy of Sciences, Beijing, 100049 China

**Keywords:** cGAS, cGAMP, innate immunity, cytosolic DNAs, DNA sensors

## Abstract

The host takes use of pattern recognition receptors (PRRs) to defend against pathogen invasion or cellular damage. Among microorganism-associated molecular patterns detected by host PRRs, nucleic acids derived from bacteria or viruses are tightly supervised, providing a fundamental mechanism of host defense. Pathogenic DNAs are supposed to be detected by DNA sensors that induce the activation of NFκB or TBK1-IRF3 pathway. DNA sensor cGAS is widely expressed in innate immune cells and is a key sensor of invading DNAs in several cell types. cGAS binds to DNA, followed by a conformational change that allows the synthesis of cyclic guanosine monophosphate–adenosine monophosphate (cGAMP) from adenosine triphosphate and guanosine triphosphate. cGAMP is a strong activator of STING that can activate IRF3 and subsequent type I interferon production. Here we describe recent progresses in DNA sensors especially cGAS in the innate immune responses against pathogenic DNAs.

## Introduction

Innate immunity provides the first defense line of host to invading microbes (Wu and Chen, 2014). The host has developed a series of pattern-recognition receptors (PRRs) to recognize and fight against pathogen-associated molecular patterns (PAMPs) that are present on microbes, such as peptidoglycans (Schwandner et al., 1999), lipopolysaccharides (LPS) (Brightbill et al., 1999; Zhang et al., 1999) and flagellin (Mizel et al., 2003). PRRs can also detect damage-associated molecular pattern molecules (DAMPs) that are derived from the host itself under stresses, including heat-shock proteins (Wick et al., 2014), HMGB1 (Bangert et al., 2016), ATP (Kouzaki et al., 2011), uric acid (Andrews, 2005), heparin sulfate (Tsunekawa et al., 2016) and DNA (Chan and Gack, 2016). The most known PRRs are the Toll-like receptor (TLR) family that are expressed on innate immune cells such as dendritic cells (DCs), macrophages and neutrophils (Yin et al., 2015). Most TLRs detect extracellular PAMPs (Gay et al., 2014). For example, TLR1 and TLR2 recognize triacylated lipoproteins from bacteria and GPI-anchored proteins from parasites (Kirschning et al., 1998). TLR2 and TLR6 detect diacylated lipoproteins from Gram positive bacteria and zymosan from fungi (McCurdy et al., 2003; Ozinsky et al., 2000). TLR4 is a receptor for LPS from Gram negative bacteria (Poltorak et al., 1998) and TLR5 recognizes flagellin from motile bacteria (Hayashi et al., 2001). Finally, TLR11 can be activated by uropathogenic *E. coli* and *T. gondii* (Lauw et al., 2005). Collectively, the above TLRs render the host to distinguish self from non-self PAMPs that are derived from invading pathogens.

Besides above extracellular PAMPs, microbes can also deliver PAMPs to the cytosol of host cells through bacterial secretion system or engulfment by cells (Saito and Gale, 2007; Wilkins and Gale, 2010). These cytosolic PAMPs are surveyed by intracellular PRRs (Beachboard and Horner, 2016). For instance, intracellular LPS can be recognized by inflammatory caspases (Shi et al., 2014; Yang et al., 2015), and intracellular flagellin is detected by NAIP5 (Gong and Shao, 2012; Zhao et al., 2011). Engulfed CpG rich DNAs are sensed by TLR9 in the endosomal compartment (Hemmi et al., 2000; Krieg, 2003). In general, all microbes rely on DNA or RNA for their basic life activities such as protein encoding, movement and proliferation (Hornung et al., 2014). These nucleic acids are potential PAMPs that are distinguished by the host (Burdette and Vance, 2013). However, this process of nucleic acid detection must be tightly regulated because of host self nucleic acids inside the cells. Improper recognition of host nucleic acids will cause autoimmune diseases (Ahn and Barber, 2014). For example, cytosolic RNAs derived from invading RNA viruses are detected by RIG-I and MDA5 in the cytosol (Kato et al., 2005; Kato et al., 2006; Weber-Gerlach and Weber, 2016; Yoneyama et al., 2004). Although there are also numerous host messenger RNAs in the cytosolic compartment, RIG-I and MDA5 respond to viral RNAs tri-phosphorylated in their 5’ ends (Leung and Amarasinghe, 2016; Lu et al., 2010; Marq et al., 2011; Wang et al., 2010). Therefore, the host develops delicate machineries to distinguish self-components from non-self-components, which is the principle of innate immune responses.

**Figure 1:**
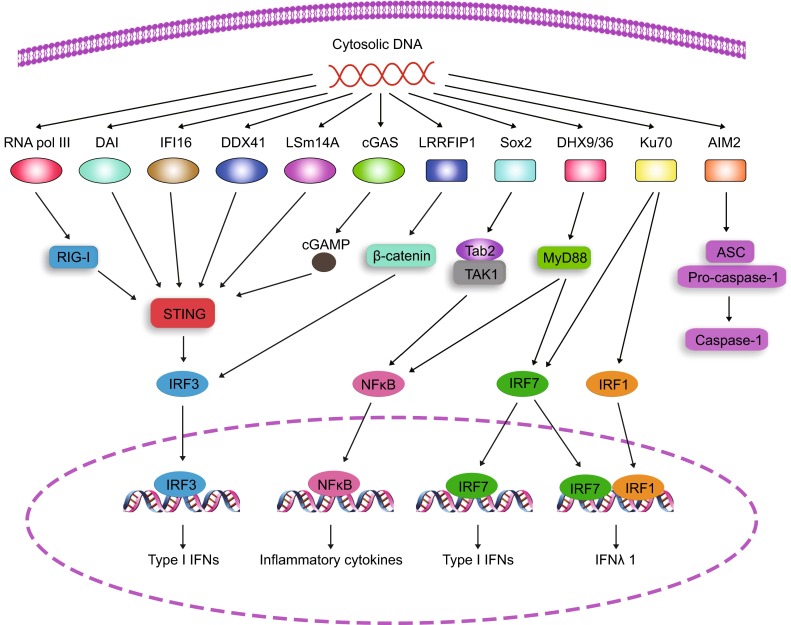
Signaling pathways of cytosolic DNA sensors with DNA challenge. Up to now, many cytosolic DNA sensors have been defined to detect intracellular double-stranded DNAs. RNA polymerase III transcribes AT-rich DNAs into RNAs that are recognized by RNA sensor RIG-I, followed by STING and IRF3 activation. DNA sensors DAI, IFI16, DDX41 and LSm14A sense dsDNA directly to activate STING for type I IFN production. In the presence of dsDNAs, cGAS catalyzes the synthesis of cGAMP, a strong activator of STING. With dsDNAs, LRRFIP1 initiates β-catenin and IRF3 activation in a STING-dependent manner. Other DNA sensors prime immune responses independently of STING. After recognition of dsDNAs, Sox2 triggers the activation of the Tab2/TAK1 complex in neutrophils. When detected by dsDNAs, DHX9/36 activates NFκB and IRF7 through MyD88. DNA sensor Ku70 triggers the activation of IRF1 and IRF7. AIM2 initiates the activation of inflammasome through ASC with DNA binding

When encountered microbial DNAs, the situation is a little complicated. Microbial DNAs do not have tri-phosphorylated groups in their 5’ ends and they are constituted by the same elements as are host DNAs (Abdullah and Knolle, 2014; Holm et al., 2013). However, eukaryotic genomic DNAs are surrounded by nuclear walls that separate DNAs from the cytosol (Beachboard and Horner, 2016). Meanwhile, invading pathogens enter the host cytosol at first and some pathogens live in the cytosol, leaving the cytosolic compartment to be contaminated by microbial DNAs (Holm et al., 2013; Hornung, 2014; Hornung et al., 2014). The cellular localization of invading DNAs provides the possibility that the host utilizes cytosolic DNA sensors to respond to these stimuli. Up to now, many cytosolic DNA sensors have been reported to recognize intracellular pathogenic DNAs (Fig. 1). For example, DDX41 (Zhang et al., 2011b), IFI16 (Orzalli et al., 2012; Unterholzner et al., 2010) and DAI (Takaoka et al., 2007) detect double stranded DNAs (dsDNAs) and activate the STING-TBK1-IRF3 pathway. LRRFIP1 binds dsDNA and triggers IRF3 activation through β-catenin (Yang et al., 2010). DHX9 and DHX36 associate with dsDNA and lead to NFκB activation through MyD88 (Kim et al., 2010). Ku70 is reported to bind dsDNA and promote production of type I interferon (IFN) through activation of IRF1 and IRF7 (Zhang et al., 2011a). AIM2 interacts with dsDNA and activates inflammasomes by recruiting ASC and pro-caspase-1 (Burckstummer et al., 2009; Fernandes-Alnemri et al., 2009; Hornung et al., 2009). Of note, Sox2 is expressed in the cytosol of neutrophils and activates the Tab2/TAK1 complex upon binding to dsDNA in a sequence-dependent manner (Xia et al., 2015).

cGAS (encoded by MB21D1 gene) has been recently defined a well-known DNA sensor that recognizes cytoplasmic DNA (Ablasser et al., 2013a; Ablasser et al., 2013b; Gao et al., 2013a; Li et al., 2013b; Schoggins et al., 2014; Sun et al., 2013; Wu et al., 2013). Double stranded DNAs (dsDNAs) longer than 36 bp are optimal for cGAS activation (Gao et al., 2013b). Once detect DNA, cGAS undergoes a conformational change that allows ATP and GTP to come into the catalytic pocket, leading to the synthesis of cGAMP, a strong activator of the STING-TBK1 axis (Civril et al., 2013; Gao et al., 2013b; Kranzusch et al., 2013; Wu et al., 2013; Zhang et al., 2014). cGAS is involved in immune recognition of various DNA viruses, certain retroviruses, and even intracellular bacteria (Collins et al., 2015; Li et al., 2013b; Wassermann et al., 2015; Watson et al., 2015; Yoh et al., 2015). cGAS can be activated by dsDNAs and DNA-RNA hybrids (Mankan et al., 2014). Besides sensing exogenous pathogenic DNAs, cGAS is also responsible for monitoring self-originated DNA (Rongvaux et al., 2014; White et al., 2014). During HSV infection, mitochondrial integrity is destroyed, causing the release of mitochondrial DNA (mtDNA) that can be detected by cytosolic cGAS (West et al., 2015). Moreover, in caspase deficient cells, mtDNA is delivered to the cytoplasm, causing cGAS activation during the process of apoptosis (Rongvaux et al., 2014; White et al., 2014). In this review, we will introduce the discovery of cGAS and describe the properties of cGAS as a DNA sensor.

## Innate immune responses induced by DNA sensors

Although TLR9 detects pathogenic DNAs in plasmacytoid dendritic cells (pDCs), there are still TLR-independent signaling pathways exiting in other cell types (Ishikawa and Barber, 2008). To search for new DNA sensors, several groups identified STING (also known as MITA, MPYS, ERIS and TMEM173) as a key player in the process of cytoplasmic DNA sensing (Ishikawa and Barber, 2008; Jin et al., 2008; Sun et al., 2009; Zhong et al., 2008). STING is essential for innate immune responses against DNA viruses, bacteria, retroviruses, certain RNA viruses and protozoan parasites (Ma and Damania, 2016; Paludan and Bowie, 2013). STING deficient cells fail to produce type I interferons post cytoplasmic DNA stimulation (Ishikawa and Barber, 2008) and STING knockout mice are extremely susceptible to herpes simplex virus 1 infection (Ishikawa et al., 2009). It thus may be plausible to consider STING as a direct DNA sensor in the cytosol. However, whether STING directly senses invading DNAs is still controversial. STING has been proposed to associate with cytoplasmic DNAs (either single-stranded or double-stranded) (Abe et al., 2013), but structural studies have not favored the hypothesis that STING binds directly to dsDNAs (Burdette and Vance, 2013). Moreover, expression of wild-type STING does not restore dsDNA responses in 293T cells that lack cellular machineries to respond to dsDNAs (Burdette and Vance, 2013; Shu et al., 2012), suggesting that there might be additional signaling factors upstream of STING (Fig. 1). Collectively, already identified DNA sensors can be classified into STING-dependent and -independent groups.

### Sting-dependent DNA sensors

DAI (DLM-1/ZBP1) had been reported to be a cytoplasmic DNA sensor that activates innate immune responses post binding dsDNA (Takaoka et al., 2007). Overexpression of DAI increases DNA-induced type I IFN production. Conversely, DAI knockdown severely inhibits DNA-initiated immune responses. DAI binds to dsDNA directly and enhances association with the transcription factor IRF3 post DNA binding. However, DAI deficient mice or cells display normal type I IFN responses stimulated by dsDNA (Ishii et al., 2008), suggesting that DAI may be dispensable for cytosolic DNA recognition. Whether DAI exerts its DNA sensing role in particular cell types still remains elusive.

Through a cell fractionation system, RNA polymerase III was identified as a cytosolic DNA sensor for poly(dA:dT) DNAs (Ablasser et al., 2009; Chiu et al., 2009). In the cytosol, RNA polymerase III converts poly(dA:dT) to RNA with 5’ triphosphorylation. The converted 5’-ppp RNA then initiates the RIG-I-MAVS pathway and NFκB activation in human and mouse cells. However, macrophages or DCs from MAVS deficient mice are still able to produce type I interferons post poly(dA:dT) stimulation (Ablasser et al., 2009). Poly(dA:dT)s are DNA molecules that activate other DNA sensing pathways such as the cGAS-STING pathway or IFI16 pathway. That could be one of the possibilities for remaining type I interferon responses in MAVS deficient mice.

The pyrin and HIN domain-containing (PYHIN) protein IFI16 is another DNA sensor that associates with viral DNAs directly upon viral infection, followed by recruitment of STING and activation of the innate immune response (Unterholzner et al., 2010). The role of IFI16 in cytosolic DNA sensing is still under debate. IFI16 is a nuclear protein albeit it can be detected in the cytoplasm. IFI16 was originally reported to bind viral DNA in the cytoplasm (Unterholzner et al., 2010). It was latterly defined to be able to discern viral genomic DNAs in the nucleus (Orzalli et al., 2015). IFI16 knockdown inhibits IRF3 and NFκB activation upon detection of viral DNAs, but the knockdown effects of IFI16 were not repeated well among different studies. A mouse model with complete IFI16 knockout will be needed to solve this dispute.

In myeloid DCs (mDCs), DDX41, a member of the DEXDc helicase family, was identified as an exogenous DNA sensor (Zhang et al., 2011b). DDX41 knockdown inhibits type I IFN and cytokine production post cytoplasmic DNA stimulation in mDCs. DDX41 and STING overexpression enhances IFN production. Moreover, DDX41 binds DNA and colocalizes with STING in the cytoplasm. DDX41-mediated innate immune responses depend on TBK1-IRF3 and NFκB activation. Interestingly, DDX41 associates with STING in the steady state, even without challenging with cytoplasmic DNA, suggesting that DDX41 may detect exogenous DNA and trigger the STING-IRF3 signaling pathway very rapidly.

LSm14A, a member of the LSm family, has been defined to be responsible for immune sensing of viral DNAs or RNAs (Li et al., 2012). LSm14A directly binds to DNAs and RNAs. LSm14A knockdown hinders the production of type I IFNs post cytosolic DNA or RNA challenge. Moreover, LSm14A quickly responds to exogenous nucleic acids and leads to early-phase production of IFNs. When LSm14A binds to foreign DNA, it recruits STING for IRF3 activation. While binding viral RNA, LSm14A colocalizes with RIG-I-MAVS and activates IRF3 through the RIG-I-MAVS signaling pathway.

### Sting-independent DNA sensors

Via mass spectrometry, aspartate-glutamate-any amino acid-aspartate/ histidine (DExD/H)-box helicase 9 (DHX9) and DHX36 have been determined to bind CpG DNAs in pDCs (Kim et al., 2010). DHX36 binds CpG-A and DHX9 binds CpG-B, respectively. Moreover, DHX36 is required for IRF7 activation upon CpG-A stimulation, while DHX9 is essential for NFκB activation post CpG-B challenge. Of note, DHX9 and DHX36 are localized in the cytosol and associate with MyD88, a key player involved in the innate immune signaling.

Through siRNA-based screening, LRRFIP1 was identified as a cytoplasmic DNA sensor. LRRFIP1 knockdown leads to inhibition of type I IFN production upon *L. monocytogenes* infection (Yang et al., 2010). Moreover, LRRFIP1 binds non-self nucleic acids such as dsRNAs and dsDNAs. LRRFIP1 then recruits and activates β-catenin that binds to the C-terminal part of IRF3. β-catenin also recruits the acetyltransferase p300 to IRF3 and promotes the activation of the IFN-β enhanceosome. However, LRRFIP1 mediated IFN production bypasses STING, a critical player in the DNA-triggered type I interferon pathways. The exact role of LRRFIP1 in the innate immune responses still needs to be further verified through genetic knockout models.

Except for type I IFNs, type III IFN has been also reported to be generated with foreign DNA stimulation in innate immune cells (Zhang et al., 2011a). Through pull-down assays and knockdown studies, a DNA repair protein Ku70 was identified as a cytoplasmic DNA sensor. Ku70 is expressed throughout a cell. Upon binding foreign DNA, Ku70 activates IRF1 and IRF7, both of which translocate to the nucleus and associate with the promoter of IFN-λ1. Unlike other DNA sensors, Ku70 recognizes DNAs with a longer sequence (usually longer than 500 bp). It is hypothesized that more than one Ku70 on target DNAs are required for proper activation of IRF1 and IRF7. In addition, Ku70 overexpression in the cytosol does not activate IFN-λ1, suggesting that some unknown factors may be needed for Ku70-mediated immune responses.

Besides induction of type I IFNs and inflammatory cytokines, cytoplasmic DNAs also induce the activation of inflammasomes that promote the maturation of IL-1β through caspase-1. Inflammasome components NLRP3 and ASC (apoptosis-associated speck-like protein containing a caspase activation and recruitment domain) were identified as DNA sensors (Muruve et al., 2008). Engulfed adenoviral DNA is detected by NLRP3 and ASC, leading to maturation of pro-IL-1β in macrophages. Mice deficient of NLRP3 or ASC impair innate inflammatory responses when challenged with adenovirus particles. The inflammasome can also be activated by cytosolic DNAs originated from cytoplasmic bacteria, viruses or even leaking host genomic DNAs. These cytoplasmic DNAs are recognized by ASC but not NLRP3, suggesting that the inflammasome components may have the ability to discern disparities among DNAs with different origins. By searching the PFAM database, a PYHIN family member protein absent in melanoma 2 (AIM2) was identified as an intracellular DNA sensor that is essential for caspase-1 activation (Burckstummer et al., 2009; Fernandes-Alnemri et al., 2009; Hornung et al., 2009). AIM2 binds dsDNA in a sequence-independent manner through its HIN200 domain. AIM2 then recruits ASC via its pyrin domain, leading to caspase-1 and NFκB activation. AIM2 knockdown suppresses caspase-1 activation upon dsDNA stimulation.

### Mysteries prior to cGAS discovery

As mentioned above, many DNA sensors have been defined to mediate recognition of exogenous DNAs. However, most of them rely on STING to initiate the production of type I IFNs. STING plays a central role in innate immune responses against foreign DNAs. Structural analysis shows that STING does not have the cavity to bind dsDNAs. Interestingly, STING has a high affinity for cyclic dinucleotides such as c-di-GMP or c-di-AMP. Cyclic dinucleotides are important secondary messenger molecules involved in signaling of bacteria. They are secreted into the cytosol of host during the invasion or replication of bacteria by multidrug efflux pumps (Whiteley et al., 2015). c-di-AMP secreted by *L. monocytogenes* activates the STING-IRF3 pathway, leading to elevated type I interferon levels that hinder the clearance of intracellular bacteria (Auerbuch et al., 2004; O’Connell et al., 2004). It is curious that STING has such a high affinity for cyclic dinucleotides which seems irrelevant to exogenous DNA recognition.

Many genes involved in innate immune responses are interferon-stimulated genes (ISGs) that are upregulated post infection. Through large-scale ISG screening, six important ISGs were identified as below: IRF1, RIG-I, C6orf150 (also known as cGAS, encoded by MB21D1 gene), IFITM3, HPSE and MDA5 (Schoggins and Rice, 2011; Schoggins et al., 2011). Among these ISGs, C6orf150 and HPSE were less investigated with unknown functions in the antiviral process. Meanwhile, whether these ISGs play an essential role in foreign DNA recognition remained elusive.

### cGAS and cGAMP

Based on activity assays using cell extracts, cyclic guanosine monophosphate–adenosine monophosphate (cGAMP) was identified to be an activator of STING (Wu et al., 2013). Mammalian cytoplasmic extracts catalyze the synthesis of cGAMP from adenosine triphosphate (ATP) and guanosine triphosphate (GTP) in the presence of dsDNAs. Both transfected dsDNA and viral DNA initiate the synthesis of cGAMP that binds to STING and initiates IRF3 activation and interferon production. Through fractionation of cytosolic extracts, cGAMP synthase (cGAS) was identified as the missing link between foreign dsDNA and cGAMP (Sun et al., 2013). cGAS, a member of the nucleotidyltransferase family, binds to dsDNA directly in the cytosol and catalyzes cGAMP synthesis from ATP and GTP (Fig. 1). cGAS overexpression promotes IRF3 activation and subsequent type I IFN production. In contrast, cGAS knockdown suppresses the IRF3 activation and type I IFN generation in a STING-dependent manner. In addition to transfected dsDNAs and viral DNAs, cGAS also mediates immune responses against retroviruses such as HIV (Gao et al., 2013a). HIV infection triggers the production of cGAMP through cGAS. HIV reverse transcriptase knockdown abolishes cGAMP production and type I IFN generation, suggesting that the transcriptase is essential for cGAS-mediated immune responses. Indeed, cGAS recognizes reversed transcribed DNAs from HIV RNAs, providing an important host sensor for retroviruses. Soon afterwards, the DNA sensing role of cGAS was validated by genetic knockout models (Li et al., 2013b). cGAS knockout mice abolish the ability to initiate type I IFN production when administrated with cytosolic DNAs either through transfection or viral infection. In addition, cGAS deficient innate immune cells such as macrophages, DCs or fibroblasts do not respond to foreign DNAs. Consequently, cGAS deficient mice are more susceptible to DNA viruses.

As a nucleotidyltransferase, cGAS catalyzes the synthesis of a product that is totally different from cyclic dinucleotides characterized previously. cGAS catalyzes the synthesis of cGAMP that has a 2′-5′ and a 3′-5′ phosphodiester linkage ([Gp(2′-5′)Ap(3′-5′)]) (Ablasser et al., 2013a; Gao et al., 2013b). cGAS promotes the synthesis of cGAMP from ATP and GTP through two steps (Ablasser et al., 2013a). cGAS first catalyzes the synthesis of a 2′-5′-linked dinucleotide from ATP and GTP, followed by another catalysis of a 3′-5′ phosphodiester linkage that finally cyclizes ATP and GTP. During the first step of the synthesis, the attacking nucleotide determines the phosphodiester bond type generated from ATP and GTP. If 5′-GTP is the attacking nucleotide, a 2′-5′-linkage will be formed. If 5′-ATP is the attacking one, a 3′-5′-linkage will be generated. However, cGAS prefers GTP as the attacking nucleotide, generating only linear pppGp(2’-5’)A. This feature of cGAS results in almost absolute synthesis of [Gp(2′-5′)Ap(3′-5′)] in the second step of cGAMP production. This 2′-5′-linked dinucleotide is a strong agonist of STING and binding of cGAMP to STING leads to STING conformational change and subsequent IRF3 activation (Gao et al., 2013b). STING forms a “closed” conformation from a “open” conformation once binds to cGAMP (Zhang et al., 2013). Structural analyses show that STING exists as a symmetrical dimer with or without binding to its ligands. The STING dimer forms a V-shaped conformation with a pocket between two STING molecules. Ligand binding causes STING conformation change with an inward-shift of the symmetrically-related α2-hilices on STING. For human STING protein, positions 230 and 232 are essential for best responses to 2’-5’-linked cGAMP. Substitution of amino acid 232 impairs the immune responses triggered by c-di-GMP but not cGAMP.

Like many secondary messengers, cGAMP functions are not limited to the cell where it is produced (Ablasser et al., 2013b; Chen et al., 2016). In human and mouse cells, cGAMP is transferred from cells to their neighbors by gap junctions, resulting in STING activation and type I IFN production in neighboring cells (Ablasser et al., 2013b). This paracrine-like activation of neighboring cells provides a quicker response to danger DNA signals than type I IFNs or cytokines do. Furthermore, cGAMP transfer requires less host protein machineries that may be inhibited by viral factors, providing the host more opportunities to produce type I IFNs or cytokines to alert the host for viral infections. cGAMP can also be transferred between cells through viral packaging (Bridgeman et al., 2015; Gentili et al., 2015). In virus infected cells, cGAMP is packaged into the viral particles, or even the extracellular vesicles. These cGAMP containing viruses trigger the activation of STING pathway once they release their contents in the host cytosol, providing a very fast recognition of invading viruses for the innate immune responses. In fact, cell-free viruses do contain cGAMP (Gentili et al., 2015). cGAMP transfer also plays an important role in tumor metastasis (Chen et al., 2016). Tumor cells such as breast and lung cancer cells can also deliver the secondary messaging molecule cGAMPs to target cells once they form gap junctions with astrocytes, the most abundant cells in the brain. Tumor cells express protocadherin 7 that promotes the formation of gap junctions with the help of connexin 43. Astrocytes activated by cGAMP start to produce inflammatory cytokines through the STING pathway, leading to STAT1 and NFκB activation in brain metastatic cells and eventually supporting the growth and chemoresistance of tumor cells.

### Structural basis of cGAS activity

**Figure 2:**
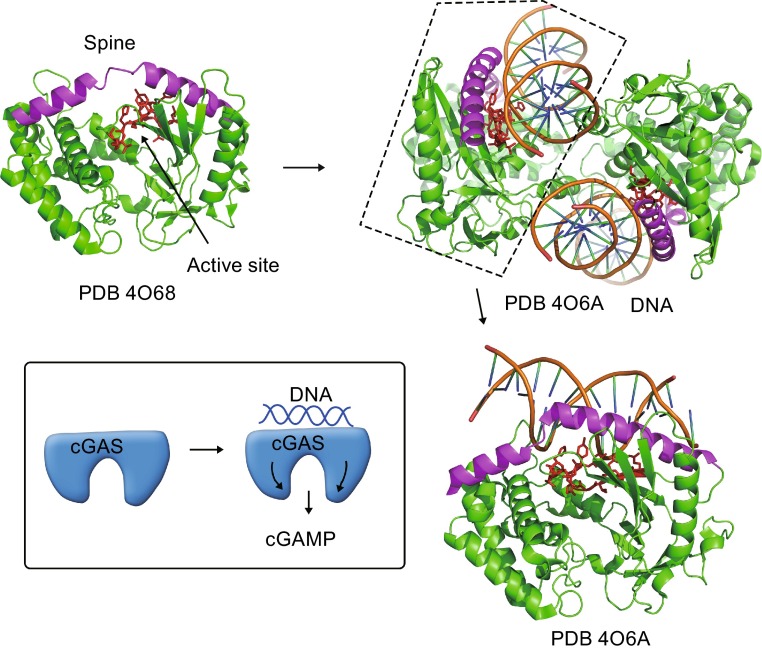
Structural basis of cGAS binding to DNA. In a resting state, cGAS exists with a monomer form. The DNA binding site comprises helices that form a flat spine. Post DNA binding, cGAS forms dimerization and undergoes conformational changes that render the spine region twisted, promoting synthesis of cGAMP. *Inset* is an illustration of cGAS activation upon binding DNA

Structural analyses provide a better understanding of how cGAS works in the process of DNA recognition. Mouse cGAS has an NTase domain from amino acid 148 (aa148) to aa370 and a Mab21 domain from aa199 to aa498 (Sun et al., 2013). The Mab21 domain is composed of two lobes divided by a deep cleft (Civril et al., 2013). One lobe has the NTase domain with a twisted β-sheet flanked by two α-helices (Fig. 2). Along the cleft, two β-sheets harbor the catalytic residues. Opposite of the catalytic site is a fairly flat surface that compromises the nucleotide-binding loop (Fig. 2). A protrusion is located on the edge of the surface, comprising conserved histidine and cysteines. This is a Zn^2+^ ion binding loop denoted as “Zn thumb” (Fig. 2). Functional analyses with cGAS mutations show that the active site residues (human G212, S213, E225 and D227) are required for the synthase activity of cGAS. Amino acids on the nucleotide-binding loop (K173, R176, K407 and K411) are essential for DNA binding. K173A and R176A mutations of human cGAS reduce cGAS activity (Civril et al., 2013) and R158E mutation of mouse cGAS fails to induce cGAMP synthesis (Li et al., 2013a). In a resting state, cGAS can associate with nucleotides such as UTP. However, UTP binding does not cause significant conformational change of cGAS. When cGAS binds dsDNAs, a tremendous structural switch is induced in the spine helix of cGAS, resulting in a “close” conformation of cGAS lobes and subsequently reformed active sites.

cGAS dimerization is also an important prerequisite for cGAS activation (Li et al., 2013a; Zhang et al., 2014). cGAS exhibits an autoinhibited conformation that the activation loop directs to the outside, hindering the synthesis of cGAMP from ATP and GTP (Fig. 2). However, when cGAS binds to dsDNA, the positively charged residues on the nucleotide-binding loop associate with negatively charged nucleotide residues, leading to conformational changes that allow cGAMP catalyzation. Of note, cGAS is a monomer in the absence of dsDNAs. When cGAS binds to dsDNA, a 2:2 dimer is formed between cGAS and dsDNA (Li et al., 2013a; Zhang et al., 2014). Residues N389 to E398 in the Zn thumb are essential for cGAS dimmer formation. Moreover, residues A346 and K347 also favor dimer formation through forming two hydrogen bonds with residue E398. The dimerization of cGAS seems conserved among species since residues K347, K394 and E398 that promote dimer formation are highly conserved in different species.

### cGAS mediates immune responses

**Figure 3:**
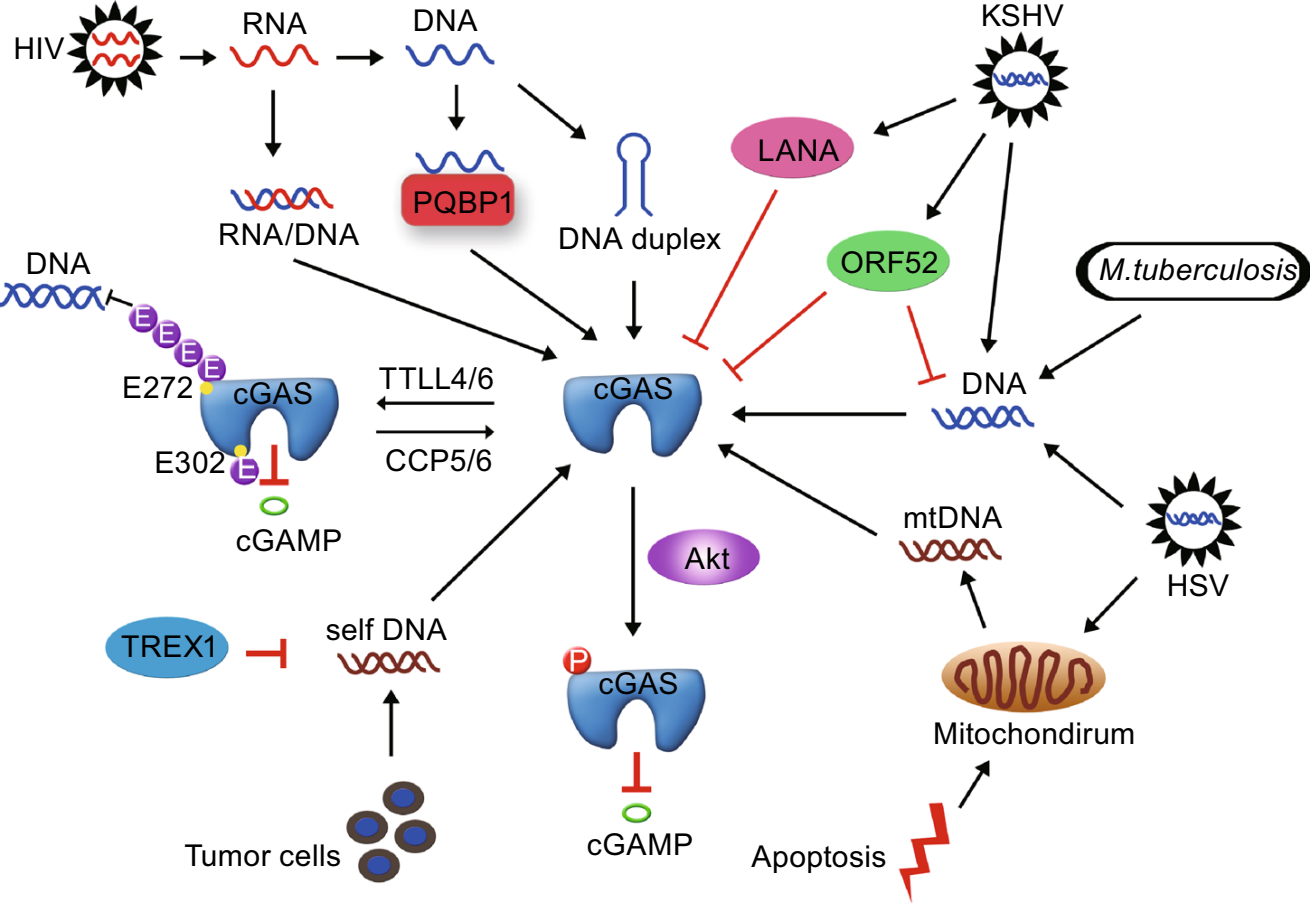
cGAS mediates dsDNA recognition and its regulation. cGAS is essential for immune recognition of foreign DNAs from various sources, such as DNA viruses, retroviruses and intracellular bacteria. cGAS activity is tightly regulated to prevent excessive activation. Besides host modifiers of cGAS, microbes possess various molecules to inhibit cGAS activity for their survival

cGAS is essential for immune recognition of foreign DNAs from various sources, such as DNA viruses, retroviruses and intracellular bacteria (Fig. 3) (Collins et al., 2015; Gao et al., 2013a; Wassermann et al., 2015; Watson et al., 2015; Yoh et al., 2015). The role of cGAS in HIV infection has been intensely investigated post cGAS discovery. There are two types of HIVs in humans: HIV-1 and HIV-2. Compared with HIV-2, HIV-1 is more common and pathogenic. Besides infected T cells, HIV-2 also replicates in DCs accompanied by activated innate immune responses (Manel et al., 2010). Further analysis shows that cellular factor SAMHD1 restricts HIV-1 infection in DCs and macrophages through negative regulation of interferon responses (Hrecka et al., 2011; Laguette et al., 2011). However, HIV-2 encodes Vpx that mediates SAMHD1 degradation through the CRL4-DCAF1 E3 ubiquitin ligase, resulting in HIV-2 replication in DCs and macrophages (Hrecka et al., 2011; Laguette et al., 2011). DCs sense reverse-transcribed viral cDNA of HIV-2 by cGAS, leading to type I IFN production and surrounding T cell activation. HIV-1 encoded capsid protects its viral cDNA from cGAS-mediated sensing in DCs (Lahaye et al., 2013). DCs play an essential role in immune responses against HIVs. Co-infection of HIV-1 and HIV-2 leads to a delayed progression of AIDS compared with HIV-1 infection alone, suggesting a positive role of HIV-2 infection in DCs in controlling HIV infection (Esbjornsson et al., 2012).

During the reverse transcription of HIV viral RNAs, only single-stranded DNA is generated. However, based on previous knowledge on cGAS, only double-stranded DNA can associate with and activate cGAS (Wu and Chen, 2014). Then how HIV-derived DNA is recognized by cGAS? Actually, HIV-derived single-stranded DNA forms short base-paired DNA stretches that are strong activators of cGAS (Herzner et al., 2015). These short dsDNAs (also called Y-form DNA) formed from single-stranded DNAs are the predominant viral DNAs in the cytosol during the early infection of host cells. Y-form DNAs are characterized by unpaired guanosines flanking the stem region. They are flexible in respect of the numbers of unpaired guanosines and represent a newly discovered robust PAMP.

Besides forming Y-form DNAs, HIV viral DNAs are also recognized by a cellular polyglutamine binding protein PQBP1 (Yoh et al., 2015). Reverse-transcribed viral DNAs are directly bound by PQBP1 that interacts with cGAS to trigger the STING-IRF3 signaling pathway. PQBP1 knockdown greatly diminishes HIV-induced immune responses in human DCs. PQBP1 binds viral DNAs through its C-terminal domain and interacts with cGAS through its N-terminal WW domain. Moreover, DCs with PQBP1 mutation attenuates immune responses against HIV. However, the exact role of cGAS in HIV infection and AIDS progression is still elusive. IFI16 has been reported to be a DNA sensor for HIV viral DNAs in CD4 T cells (Monroe et al., 2014). During AIDS progression, quiescent CD4 T cells have accumulated reverse-transcribed viral DNAs in their cytosol and are constantly depleted. Viral DNAs are detected by IFI16 that leads to IFN production, caspase-1 activation and consequently pyroptosis of host cells. Interestingly, cGAS is not present in CD4 T cells, indicating cell type-restricted expression patterns of DNA sensors. Recently, both cGAS and IFI16 have been reported to be required for type I IFN induction in human fibroblasts infected by HSV (Orzalli et al., 2015). In these cells, cGAS produces cGAMP post HSV infection but the amount of cGAMP is quite low. Partial cGAS is located in the nucleus where it interacts with nuclear IFI16 and stabilizes IFI16. In the nucleus, IFI16 but not cGAS directly binds to viral DNAs, followed by cytoplasmic translocation and STING activation. However, how IFI16 discriminates viral DNA from host genomic DNA in the nucleus is still not clear.

cGAS also senses DNAs from DNA viruses. cGAS can recognize HSV genomic DNA upon infection, triggering type I IFN production (Li et al., 2013b). Innate immune cells such as macrophages, dendritic cells and fibroblasts are unable to produce type I IFNs in response to HSV infection. Moreover, cGAS deficient mice are more susceptible to HSV infection. Moreover, cGAS is also responsible for sensing DNAs from KSHV and HPV viruses (Lau et al., 2015; Ma et al., 2015).

cGAS plays an important role in monitoring bacterial DNAs. As a classic intraphagosomal pathogen, *M. tuberculosis* can translocate from phagolysosomes to cytoplasm where *M. tuberculosis* PAMPs associate with host cytosolic sensors (van der Wel et al., 2007). *M. tuberculosis* activates the cytoplasmic DNA surveillance pathway through disrupting phagosomes by proteins of the secretion system such as ESX-1, ESAT-6 and CFP-10 (De Leon et al., 2012; Manzanillo et al., 2012; Simeone et al., 2012). Microbial DNAs then leak out to the cytosol where they are recognized by cGAS, followed by STING-IRF3 activation (Collins et al., 2015; Wassermann et al., 2015; Watson et al., 2015).

In addition to exogenous DNAs, self-DNAs are also sources of activators that initiate type I interferon production through cGAS. In cells undergoing apoptosis, mitochondrial DNA is released by Bak and Bax. The leaking DNA is then detected by cGAS, followed by STING-IRF3 activation and type I IFN production. However, apoptotic caspases inhibit type I IFN production (Rongvaux et al., 2014; White et al., 2014). The elevated type I IFN production is only observed in caspase deficient or inhibited apoptotic cells. How caspases block mitochondrial DNA-induced cGAS activation is not clear yet. It may be caused by one of the molecules involved in cGAS-STING signaling that is cleaved by apoptotic caspases during apoptosis. For example, IRF3 is a target of caspases (Crawford et al., 2013). Apoptotic caspases may inhibit DNA release from the mitochondrium. Another possibility might be that mitochondrial DNA is degraded by nucleases which are activated by apoptotic caspases. Caspase-mediated inhibition of mitochondrial DNA-induced type I IFN production is common among distinct cell types such as hematopoietic stem cells (White et al., 2014) and mouse embryonic fibroblasts (Rongvaux et al., 2014). It will be intriguing to test whether cGAS itself may be a direct target of caspases. Similar to mitochondrial DNA release during apoptosis, mitochondrial DNAs also leak out to the cytosol during aging or viral infections (West et al., 2015). In cells deficient of *Tfam*, an essential protein for mitochondrial DNAs stability, mitochondrial DNAs leak out to the cytosol due to aberrant packaging of mitochondrial DNAs. The leaked mitochondrial DNAs then activate the cGAS-STING-IRF3 signaling pathway and finally cause type I IFN production. Moreover, viral infection also results in mitochondrial DNA release (West et al., 2015). Herpesvirus infection causes mitochondrial DNA stresses in infected cells and gives rise to type I IFN production. Therefore, at least two means of innate immune responses are utilized by the host to counteract viral infection. One means is the canonical viral DNA recognition mediated by cGAS. The other is recognizing mitochondrial DNA caused by viral infection.

Besides mitochondrial DNAs, recognition of self-genomic DNAs by cGAS causes autoimmune diseases. TREX1 is a cytoplasmic exonuclease that degrades DNA either from self or engulfment (Crow et al., 2006; Morita et al., 2004; Yang et al., 2007). During DNA repair of damages caused by endogenous or environmental stresses, short single-stranded DNAs are continuously generated (Lindahl and Wood, 1999). These single-stranded DNAs leak out to the cytoplasm, where they are drawn back into the nucleus by DNA repair and replication factors such as RPA and Rad51 (Wolf et al., 2016). RPA or Rad51 knockdown causes accumulation of single-stranded DNAs in the cytosol. TREX1 is anchored on the outer nuclear membrane to degrade leaking DNAs immediately. RPA or Rad51 inhibition leaves more DNAs in the cytosol than TREX1 can degrade. Similarly, TREX1 deficiency exhausts the DNA-drawing role of RPA and Rad51, resulting in accumulation of DNAs in the cytoplasm. In TREX1 deficient mice, accumulated self-DNAs activate cGAS for cGAMP production, followed by type I IFN and inflammatory cytokine production (Ablasser et al., 2014; Gao et al., 2015; Gray et al., 2015). Persistent cytokine production leads to autoimmune diseases in the host. A further knockout of cGAS in the TREX1 deficient mice reduces type I IFN and cytokine production, alleviating the autoimmune symptoms. Selective inhibition of the cGAS-mediated DNA sensing pathway sheds new lights on therapies of autoimmune diseases.

In neuroinflammatory autoimmune diseases such as Aicardi-Goutières syndrome (AGS), constant mutations in nucleotide-processing proteins are observed (Gunther et al., 2015). More than 50% of AGS patients have mutations in genes encoding RNase H2 components such as RNase H2A, RNase H2B and RNase 2C (Crow et al., 2015). RNase H2 is the main RNase H enzyme that is essential for ribonucleotides removal during replication (Cerritelli and Crouch, 2009; Nick McElhinny et al., 2010). A point mutation in RNase H2A (G37S) causes catalytic disability and accumulated RNA/DNA hybrids, followed by increased expression of ISG genes. STING deficiency partially rescues the phenomenon caused by RNase H2A mutation (Pokatayev et al., 2016).

cGAS-mediated type I IFN production is also essential for tumor surveillance. In tumor microenvironments, spontaneous T cell activation is constitutively observed with signatures of production of cytokines such as type I IFNs by CD8α^+^ DCs (Diamond et al., 2011; Fuertes et al., 2011). However, in STING deficient mice, T cell responses are abolished, suggesting that the foreign DNA recognition pathway might be involved (Woo et al., 2014). When tumor cells are engulfed by antigen-presenting DC cells, tumor-derived DNAs are detected by cytoplasmic cGAS in DC cells. The recognition of tumor DNAs in the cytoplasm results in type I IFN production in DC cells and subsequent T cell activation. Even with this IFN-mediated immune surveillance, transplantable tumor cells continue to grow in most cases. Tumor cells have developed immune escape mechanisms to inhibit the innate immune responses in the tumor microenvironment (Gajewski et al., 2013; Spranger et al., 2013). Therefore, immunotherapies that aim to enhance the type I IFN production in the tumor microenvironment might provide approaches for cancer therapy. In addition to immunotherapies, radiotherapy also utilizes the cGAS-STING pathway to enhance T cell activities (Deng et al., 2014). Radiation causes DNA damage in tumor cells, facilitating DNA presenting in DC cells. Tumor DNAs then activate the cGAS-STING pathway and type I IFN production in DCs, resulting in intense priming of T cells. These observations explain how type I IFNs are produced post local radiation (Burdette and Vance, 2013).

The cGAS-cGAMP signaling has also immune adjuvant effects (Carroll et al., 2016; Li et al., 2013b). cGAMP serves as an adjuvant that accelerates activation of antigen-specific T cells in mice. Intramuscular administration of ovalbumin (OVA) and cGAMP significantly enhances OVA-specific antibody production (Li et al., 2013b). Immunized CD4 and CD8 T cells upregulate levels of IL-2 and IFN-λ post stimulation with OVA peptides. Besides cGAMP, another molecule cationic polysaccharide chitosan displays adjuvant effects that signal through the cGAS-cGAMP pathway (Carroll et al., 2016). Cationic polysaccharide chitosan causes mitochondrial ROS generation, followed by mitochondrial DNA release to the cytosol. Mitochondrial DNAs are then detected by cGAS, followed by cGAMP production and type I IFN generation in DCs. Therefore, type I IFNs promote the maturation of DCs and subsequently promotes enhanced cellular immune activities.

### Regulation of cGAS activity

Like many immune responses, cGAS activity is tightly regulated to prevent excessive activation (Fig. 3). Protein post-translational modifications (PTMs) such as phosphorylation, acetylation, glycosylation, and ubiquitination play important roles in the regulation of activities of target proteins by changing their chemical or structural properties. In mice deficient of cytosolic carboxypeptidases CCP5 and CCP6, immune responses against DNA viruses are severely compromised (Xia et al., 2016). Macrophages isolated from these mice impair activation of IRF3 post DNA viral infection. cGAS is mono- and poly-glutamylated in macrophages by TTLL4 and TTLL6, respectively. cGAS is poly-glutamylated by TTLL6 at Glu272 where the poly-glutamylated glutamate chains are removed by CCP6. In parallel, cGAS is also mono-glutamylated by TTLL4 at Glu302 where the mono-glutamylated glutamate is removed by CCP5. Poly-glutamylated chains hinder the association between cGAS and dsDNAs, while mono-glutamylated glutamate abolishes the DNA synthesis activity of cGAS. These results uncover an essential role of cytosolic carboxypeptidases (CCPs) and tubulin tyrosine ligase-like enzymes (TTLLs) in the antiviral events through modifying the cytosolic DNA sensor cGAS, which might shed new lights on clinical treatment of diseases caused by DNA viruses. Besides glutamylation, cGAS activity is also regulated by phosphorylation (Seo et al., 2015). During viral infections, Akt becomes activated and phosphorylates the enzymatic domain of cGAS on Ser291 (for mouse) and Ser305 (for human). Phosphorylated cGAS loses its enzymatic activity to synthesize cGAMP, leading to reduced immune responses against DNA viruses. These post-translational modifications of cGAS provide essential approaches to modulate cGAS activities during innate immune responses.

Autophagy is also involved in the regulation of cGAS activity. During the process of cGAS-mediated immune responses, cGAS associates with Beclin 1, a key regulator of autophagy (Liang et al., 2014). The direct interaction between cGAS and Beclin 1 suppresses cGAS activity and blocks cGAMP synthesis. Moreover, cytoplasmic DNAs are also degraded through the autophagy degrading system.

Besides host modifiers of cGAS, microbes possess various molecules to inhibit cGAS activity for their survival (Fig. 3). Virus-derived proteins such as E7 from HPV and E1A from adenovirus have conserved LXCXE motifs (McLaughlin-Drubin and Munger, 2009) that are required for efficient binding to STING. These proteins bind to STING and inhibit the activity of STING (Lau et al., 2015). Moreover, vIRF1 from Kaposi’s sarcoma-associated Herpesvirus (KSHV) also binds to STING, preventing the interaction between STING and TBK1 (Ma et al., 2015). CdnP, an ectonucleotidase encoded by group B streptococcus, degrades c-di-AMP secreted by bacteria, alleviating host immune responses (Andrade et al., 2016). Above findings suggest that microbes utilize a universal way to suppress cGAS activity through blocking STING activity either directly or indirectly. Microbes also produce products to inhibit cGAS activity directly (Ma and Damania, 2016). KSHV-encoded tegument protein ORF52 inhibits cGAS activity by directly binding to cGAS, abolishing the DNA binding ability of cGAS (Wu et al., 2015). Moreover, ORF52 also binds to viral DNAs directly, preventing them from being detected by cGAS. As a latency-associated nuclear antigen, KSHV-encoded LANA also exists in the cytoplasm of infected cells (Kedes et al., 1997). Cytosolic LANA directly binds to cGAS, leading to inactivaiton of cGAS and diminished expression of type I IFNs (Zhang et al., 2016).

## Concluding remarks

Although cGAS plays an important role of cytosolic DNA sensing in cell types such as macrophages, DCs and fibroblasts, there are also some cell types do not express cGAS but have normal cytosolic DNA sensing potential. For instance, in CD4 T cells, IFI16 is the main cytoplasmic DNA sensor for HIV reverse-transcribed DNAs (Monroe et al., 2014). In neutrophils, Sox2 acts as a DNA sensor for invading cytosolic bacterial DNA (Xia et al., 2015). Sox2 localizes in the cytoplasm of neutrophils and recognizes microbial DNA through its HMG domain. Moreover, Sox2 associates with TAB2 upon bacterial DNA stimulation and activates the TAB2-TAK1 kinase complex through dimerization. Interestingly, Sox2-mediated DNA recognition is sequence-dependent. Sequence-specific recognition of exogenous DNAs has long been used by low forms of life such as eubacteria and archea. Restriction endonucleases are DNA sequence-specific enzymes that are utilized by bacteria to cleave invading viral DNAs (Jinek et al., 2012). These enzymes provide the innate defense against phage DNAs. In addition, the recently developed CRISPR/Cas9 technology is also originated from bacteria. Bacteria synthesize guiding RNAs from previous integrated virus DNAs to target and degrade incoming virus DNAs (Sampson et al., 2013). This sequence reliable system is a kind of acquired immunity which mimics adoptive immunity in higher organisms. Therefore, it is plausible that higher organisms might reserve these sequence-specific defense systems during evolution, just like the DNA sensing by Sox2 in human and mouse neutrophils.

As mentioned above, cytosolic sensing of PAMPs especially pathogenic DNAs is an intensely investigated topic. The discoveries of DNA sensor cGAS and the secondary messenger cGAMP fill up the gaps between cytosolic DNAs and the STING-IRF3 signaling pathway. A further investigation of the cytosolic DNA sensing pathway and a better understanding of how innate immune system works will benefit our clinical applications of infection prevention and treatment.
